# Common Causes of Drug-Induced Liver Injury in 2025

**DOI:** 10.5152/tjg.2026.26361

**Published:** 2026-07-21

**Authors:** Einar S. Björnsson

**Affiliations:** 1 University of Iceland Faculty of Medicine, Reykjavik, Iceland; 2 Department of Gastroenterology, Landspitali University Hospital Reykjavik, Reykjavik, Iceland

**Keywords:** Antibiotics, checkpoint inhibitors, drug-induced liver injury, herbal and dietary supplements, infliximab

## Abstract

Drug-induced liver injury (DILI) is an important clinical condition and should be considered in the differential diagnosis of patients with elevated liver enzymes and normal hepatobiliary imaging. In previous studies of DILI, antibiotics were the most commonly implicated class of drugs. A newly recognized phenotype of cefazolin has been described in several studies. After an ultra-short duration of cefazolin, with 1 or 2 doses, a prominent cholestatic or mixed reaction develops 2-3 weeks later. This phenotype was originally described in the United States and has recently been reproduced in a study from Iceland. In a recent and important report from Türkiye, hepatocellular jaundice associated with the antibiotic ornidazole was described; 8% of affected patients required liver transplantation. In recent years, several new agents have been associated with other idiosyncratic DILI associated with prescription drugs, suggesting that the clinical landscape of DILI may be evolving. Liver injury resulting from the biological effects of drugs that modulate the immune system appears to be increasing. In a recent study from Barcelona, anticancer drugs, mainly checkpoint inhibitors, were the most frequent causes of DILI, followed by antibiotics, analgesics, and recreational drugs. Furthermore, several newly recognized hepatotoxic herbal and dietary supplements (HDSs) have been reported. These are *Polygonum multiflorum*, ashwagandha, green tea extract, turmeric, *Tinospora cordifolia*, Garcinia cambogia, and kratom. Most patients recover after discontinuation of the HDS, but acute liver failure (ALF), death from ALF, or liver transplantation has been reported in some cases.

**Main Points**

- Postmarketing detection of drugs with hepatotoxic potential has increased in recent years.
- Antibiotics have previously been identified as the most common class of drugs leading to drug-induced liver injury (DILI), but checkpoint inhibitors are now a more common cause of DILI than antibiotics.
- New herbal and dietary supplements have been associated with DILI, particularly *Polygonum multiflorum*, green tea extract, turmeric, *Tinospora cordifolia*, *Garcinia cambogia*, and kratom.
- Other drugs identified as emerging causes of DILI are ornidazole, ribociclib, and infliximab.

## Introduction

Drug-induced liver injury (DILI) is an important differential diagnosis in patients with elevated liver test results without an obvious etiology. The vast majority of patients present with acute liver injury, but chronic liver injury and cirrhosis have been reported with certain drugs, such as nitrofurantoin and amiodarone.^1^ The diagnosis can be challenging because no gold standard exists. Before a drug is implicated as the cause of the liver injury, other causes should be reasonably excluded. As in other areas of medicine, clinical judgment is essential, as the diagnostic workup may involve expensive investigations.

DILI is currently classified into 3 categories: (1) direct or intrinsic liver injury, such as that caused by paracetamol; (2) idiosyncratic DILI, the most common type, caused by antibiotics, such as amoxicillin–clavulanate; and (3) indirect DILI, most commonly associated with drugs that modulate the immune system, such as checkpoint inhibitors (CPIs) and infliximab. This terminology, which introduced a new category of indirect liver injury, was first proposed by Jay Hoofnagle of the National Institutes of Health in a review in the *New England Journal of Medicine*.^1^ This was later incorporated into the guidelines of the AASLD (American Association for the Study of the Liver) and indirect liver injury was introduced as the third mechanism of DILI.^2^ However, there is considerable overlap between idiosyncratic and indirect liver injury. Although this categorization was initially received with some skepticism, it now appears to be widely accepted by the scientific community.^2^^,^^3^

In the current review, only idiosyncratic and indirect types of DILI are discussed.

Many retrospective and prospective studies have demonstrated that antibiotics are the dominant class of drugs associated with DILI.^4^^-^^9^ In most studies from Western countries, amoxicillin–clavulanate (A-C) has been identified as the antibiotic most frequently implicated in DILI.^4^^,^^6^^,^^9^ The landscape of DILI appears to be changing in recent years. Liver injury resulting from the biological effects of drugs that modulate the immune system appears to be increasing. This trend was illustrated in a recent 5-year prospective study from Spain on the most common causes of DILI.^10^ Anticancer drugs, mainly CPIs, were the most frequent causes, followed by antibiotics, analgesics, and recreational drugs.^10^ In a prospective European DILI study conducted from 2016 to 2021, the most common causes of DILI were amoxicillin-clavulanate (12%), flucloxacillin (11%), atorvastatin (8%), nivolumab and ipilimumab (8%), infliximab (5%), nitrofurantoin (4.5%), disulfiram (2%), and ibuprofen (2%).^9^ The prospective European study (Pro-Euro-DILI) is a collaboration among researchers from Spain, England, Germany, Switzerland, Sweden, and Iceland.^9^ Liver injury due to HDSs has been more frequently reported in Asia.^11^^,^^12^ Although there are many similarities between DILI registries and prospective studies, there are also notable differences.^7^^-^^9^ Ibuprofen was the third most commonly implicated agent in the Spanish DILI registry^8^ (after A-C and anti-TBC drugs), whereas it has rarely been observed in the DILIN study^7^ or in DILI studies from Iceland.^6^ In the Spanish registry, infliximab has very rarely been observed,^8^ whereas it is among the top 10 most frequently implicated drugs in Iceland and in the DILIN study.^6^^,^^13^^,^^14^ Herbal and dietary supplements (HDSs) accounted for 16% of cases of liver injury in studies from Iceland and the United States,^6^^-^^7^ whereas they were reported less frequently in the Spanish registry.^8^ Although information on HDS intake was obtained in the Barcelona study, only 1 patient was reported to have HDS-related liver injury, attributed to an unknown energy drink.^10^ However, recreational drugs were among the most common causes of DILI in the Barcelona study, mostly cocaine and amphetamine,^10^ whereas recreational drugs have very rarely reported in other DILI studies.^4^^-^^9^ In the current review, a systematic search of PubMed was conducted for articles on DILI published from 2020 to 2026. The following search terms were used: ”drug-induced liver injury” (DILI), “hepatotoxicity,” and “common causes.” Clinical studies reporting newly identified agents were selected for inclusion in the systematic analysis.

### Antibiotics, Still a Common Cause of Drug-Induced Liver Injury 2025

As mentioned earlier, antibiotics have been the most commonly implicated class of drugs in DILI in both retrospective and prospective studies.^4^^-^^9^ In recently published studies of both hospitalized patients and outpatients, antibiotics have been shown to be the most common cause of DILI.^15^^,^^16^ Cephalosporins are widely used antibiotics, but despite their frequent use, they have, until recently, been rarely implicated as causative agents of DILI in previous studies.^6^^-^^9^ In the Spanish DILI registry, which included a total of 843 cases of DILI, only 5 cephalosporin-associated cases were reported, involving ceftriaxone (n = 3) and cefuroxime (n = 2), with no cases attributed to cefazolin.^8^ In the DILIN study, Vuppalanchi et al^17^ described cephalosporin-associated liver injury in 58 patients. Most patients developed liver injury after the administration of cefazolin, accounting for 70% of the cases, and the majority received cefazolin as a single dose for antibiotic prophylaxis.^17^ Notably, nearly a decade earlier, the DILIN group reported DILI associated with cefazolin in 19 patients after a single dose of cefazolin, with a median latency of 20 days.^18^ Because of this unusual clinical phenotype, with a single dose, could have a latency of up to 3 weeks, this was met with skepticism among many in the DILI field.^19^ In the recent cephalosporin-associated DILI study, the median duration of therapy was 1 day (range 1-15 days).^17^

Human leucocyte antigen (HLA)-A*02:01 was associated with an increased risk of liver injury (OR 2.5-2.7; *P* < .0001).^17^

A recent study from Iceland on antibiotic-induced DILI in hospitalized patients validated the DILIN findings regarding cephalosporin-induced liver injury.^15^ The most commonly implicated agent was amoxicillin/clavulanate (n = 23), occurring in 1 of 1327 users, followed by ceftriaxone (n = 8), occurring in 1 of 3779 users, and cefazolin (n = 7), occurring in 1 of 6363 users.^15^ Clinical and biochemical features of cefazolin-implicated cases were very similar to those previously reported by the DILIN group, with a latency of 20-30 days after 1 or 2 doses of cefazolin.^15^ Thus, this cholestatic phenotype associated with cefazolin use was reproduced in this study from Iceland. The reason cefazolin has not been prominently identified in other DILI studies remains unclear.^8^^,^^20^ One possible explanation is that when patients present with jaundice 1-3 weeks after an uncomplicated surgical procedure, neither the patients nor their physicians are aware of the exposure.^19^

A recent single-center study of pediatric DILI in Türkiye found that cephalosporins, together with methotrexate, were the most commonly implicated drug^21^ These were followed by carbapenem-group antibiotics (6.77%), steroids (6.77%), and mercaptopurine (5.04%).^21^ According to the authors, the choice of antibiotic was undergoing dynamic changes because of increasing numbers of resistant bacteria.

In another recent study from Türkiye, ornidazole-associated liver injury was reported.^22^ Ornidazole is widely used in China, Russia, India, Türkiye, and several other European countries. However, it has never been marketed in the United States, and no information regarding its hepatotoxicity is available in LiverTox.^23^ Ornidazole has efficacy and indications similar to those of metronidazole, which has been only rarely associated with liver injury and is classified as category C in LiverTox, indicating that it has been implicated as a suspected cause of DILI in only 4-11 published cases.^24^ The clinical and biochemical features were evident in this report, with a prominent hepatocellular pattern and, in a few patients, autoimmune features requiring corticosteroids.^22^ Most patients recovered after discontinuation of the drug; however, 8% (n = 8) required liver transplantation, and 4% (n = 4) died.^22^ Notably, all patients who required liver transplantation for DILI were female, although women comprised 80% of the study population.^22^ As in most other DILI studies, a limitation of this study was its nonpopulation-based design, making it impossible to evaluate the proportion of users at risk of developing DILI.

**Figure 1.**
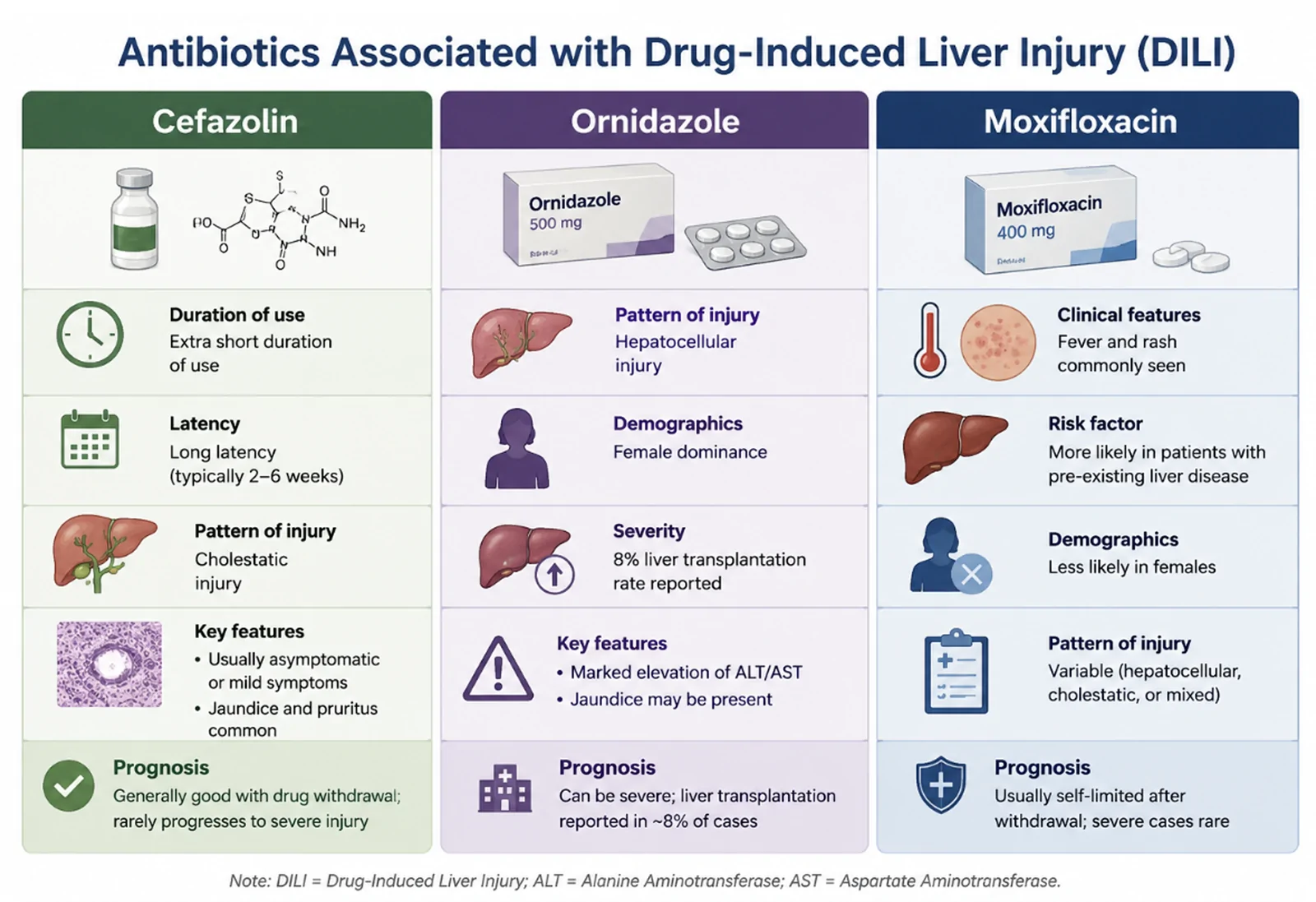
Clinical characteristics, demographic features, and outcomes of patients with drug-induced liver injury caused by 3 hepatotoxic antibiotics: cefazolin, ornidazole, and moxifloxacin.

Fluoroquinolone-induced liver injury is relatively rare.^24^ Among these agents, moxifloxacin, which is classified as category B in LiverTox, has been associated with allergic manifestations, such as fever and rash, in many reported cases.^24^ Using pharmacovigilance data from the Food and Drug Administration adverse event reporting system, a retrospective observational study evaluated suspected moxifloxacin-associated DILI in hospitalized patients.^25^ According to the study, female sex was an independent protective factor.^25^ Identified risk factors included hypoalbuminemia, hepatic comorbidities such as fatty liver, preexisting liver injury, elevated baseline alkaline phosphatase, and concomitant use of traditional Chinese medicine or respiratory medications.^25^ Although the authors identified these associations, a major limitation of this study, as with other registry-based studies, is the difficulty of establishing causality assessment when substantial data are missing regarding the exclusion of alternative causes of elevated liver test results. Figure 1 summarizes the clinical characteristics, demographic features, and outcomes of patients with cefazolin-, ornidazole-, and moxifloxacin-induced liver injury.

### Herbal and Dietary Supplements

HDSs accounted for 16% of all causes of liver injury in the Icelandic and the DILIN studies.^6^^,^^7^ A recent prospective study from Iceland aimed to identify the most common etiologies among patients with significantly elevated alanine aminotransferase (ALT) levels or concurrent elevations in ALT and alkaline phosphatase (ALP).^16^ Another aim of the study was to determine the prevalence of DILI in this patient population and identify the most commonly implicated drugs. The most common etiologies were choledocholithiasis (42%), ischemic hepatitis (17%), hepatobiliary cancer (8.4%), DILI (8.2%), and viral hepatitis (6.2%).^16^ Among patients with DILI, HDSs accounted for 8 of 54 cases (15%), most of which were associated with products containing green tea extract.^16^ Other European studies have reported similar or lower frequencies HDS-induced liver injury. In a single-center study from Türkiye, herbal medicines accounted for approximately 12% of DILI cases.^26^ *Teucrium polium* (felty germander), a traditional herbal medicine of the Labiatae family used in that region, was the most common cause of herb-induced liver injury (HILI), accounting for 7 of 10 cases.^26^ In the Spanish hepatotoxicity registry, HDSs accounted for 6% of implicated agents.^8^ Table 1 summarizes newly recognized HDSs associated with hepatotoxicity.

### Polygonum multiflorum

A systematic review from Korea identified 97 cases of HILI, of which 75% were hepatocellular.^27^ Causative agents included 21 unique herbal preparations, of which *Polygonum multiflorum* (39%) and *Dictamnus dasycarpus* (37%) were the most commonly implicated.^27^ Similar to Korea, HILI is the leading cause of *idiosyncratic* liver injury in China.^28^^,^^29^ Shen et al^28^ reported from mainland China that traditional Chinese medicines and HDSs were the most commonly implicated classes of agents, accounting for 27% of DILI cases. A recent study from China found that, among 481 patients with DILI, 63% of cases were attributed to prescription drugs and 37% to herbal products.^29^ Notably, *Polygonum multiflorum* accounted for 21% of herb-induced cases.^29^

### Ashwagandha

Ashwagandha (*Withania somnifera*), a widely used herb in Indian Ayurvedic medicine, has recently been implicated in several case reports and case series of liver injury.^30^^,^^31^ Despite claimed health benefits, there is a lack of scientific data, from well-designed and adequately performed studies, that prove its efficacy and safety. However, multiple dietary supplements containing ashwagandha are marketed in Europe and the United States. These patients typically presented with cholestatic or mixed-pattern jaundice accompanied by pruritus, with cholestatic hepatitis on histologic examination.^30,31^ The largest case series originated from India (n = 23) and reported severe outcomes, such as acute-on-chronic liver failure and liver-related death.^31^ Supplements containing ashwagandha have now been banned in Denmark, and several HDSs containing ashwagandha have also been banned in other EU countries, such as Sweden.

### Green Tea Extract

Among all ingredients used in HDSs, green tea extract has the strongest evidence for hepatotoxicity.^32^ HDSs containing green tea extract have been frequently implicated in DILI studies from different parts of the world.^6^^,^^7^^,^^11^^,^^33^ In a population-based study from Iceland, HDSs accounted for 15 of 96 DILI cases (16%).^6^ Among these 15 HDSs, 4 (27%) contained green tea *(Camellia sinensis*) extract as an active ingredient, including 2 Herbalife® products, Mega Men Heart®, and Metasys®, respectively.^6^ Similarly, in the Latin America Registry and the prospective European network (PRO-EURO-DILI-NET), HDSs containing green tea extract were frequently implicated in DILI.^9^^,^^33^ Hoofnagle et al^32^ described the clinical and genetic features of 40 patients with liver injury associated with green tea extract–containing supplements.^32^ Interestingly, these patients exhibited a distinct clinical phenotype, with a hepatocellular pattern of liver injury in 95% of cases. Moreover, 35% developed severe liver injury, and 8% required liver transplantation. Furthermore, HLA-B*35:01 was present in 72% of patients, compared with 12% of patients with DILI attributed to other drugs, a frequency similar to that observed in population controls (11%).^32^ Thus, green tea extract has very convincing biochemical and genetic hepatotoxicity potential.

### Turmeric

An increasing number of reports have described suspected turmeric-induced hepatotoxicity in recent years.^34^ In the DILIN cohort, 10 cases of liver injury were attributed to turmeric-containing supplements were identified, 90% of which exhibited a hepatocellular pattern.^34^ Half of the patients required hospitalization, and 1 died of acute liver failure (ALF). Chemical analysis confirmed the presence of turmeric in all tested products (n = 7), and some also contained black pepper. HLA-B 35:01 was identified as a risk factor for turmeric-induced HILI and was present in 70% of the patients.^34^

### Tinospora cordifolia

*Tinospora cordifolia*, commonly known as Giloy in India, is widely used in Ayurvedic medicine and has been proposed to have immune-boosting properties. During and after the COVID-19 pandemic, several cases of *Tinospora cordifolia*–associated hepatotoxicity were reported from India.^35^ The largest case series from India included 43 patients with a latency of 46 days.^35^ Notably, most patients had positive ANA at presentation and autoimmune features on liver histology.^35^ The predominant clinical phenotype was drug-induced autoimmune-like hepatitis (DI-ALH).^35^ It remains unclear whether *Tinospora cordifolia* unmasks an underlying autoimmune disorder or directly induces ALH. In a recent systematic analysis, *Tinospora cordifolia* and khat were the only HDSs associated with probable DI-ALH cases.^36^

### Garcinia cambogia

*Garcinia cambogia*, a botanical dietary supplement derived from the fruit of the tree Garcinia gummi-gutta, has been linked to hepatotoxicity in recent years.^37^ In a study from the DILIN network, 22 cases of liver injury associated with *Garcinia cambogia* were identified, including 5 attributed to *Garcinia cambogia* alone, 16 to products containing both *Garcinia cambogia* and green tea, and 1 to a product containing *Garcinia cambogia* and ashwagandha.^37^ These patients presented with hepatocellular jaundice. The frequency of the HLA-B∗35:01 allele was significantly higher among patients with *Garcinia cambogia–*containing HDSs (55%) than among patients with liver injury attributed to other HDSs (19%) or to conventional drugs (12%). The clinical and biochemical features of *Garcinia cambogia*–associated hepatotoxicity appeared indistinguishable from those of green tea extract.^37^ The association with the HLA-B∗35:01 allele suggests an immune-mediated mechanism of liver injury.^37^

### Kratom

Kratom is a herbal product used as an opium substitute, known as “ketum” in Malaysia and “kratom” in Thailand, and has been shown to have abuse potential and hepatotoxicity; it is illegal in many countries. A total of 11 cases of kratom-associated liver injury were identified in the DILIN study.^38^ Most patients were men, with a median age of 40 years, and presented with jaundice after a median latency of only 2 weeks. No patient died of liver failure or required liver transplantation.^38^ Overall, 10 of 11 patients were homozygous for the PTPN22 G allele, which has been identified as a non-HLA genetic risk factor for idiosyncratic DILI across multiple drugs.^39^

### Checkpoint Inhibitors

CPIs have revolutionized the treatment of many malignancies such as malignant melanoma and can halt the progression of the disease and keep patients in long-standing remission. CPIs target the checkpoints that can prevent immune reactions, PD ligand 1 (PDL-1), programmed cell death protein-1 (PD-1), and cytotoxic T-lymphocyte-associated protein-4 (CTLA-4). This substantial efficacy of these drugs is accompanied by a risk of immune-mediated adverse effects such as dermatitis, pneumonitis, colitis, hypophysitis, and hepatitis.^40^ This mechanism is thought to reduce immune tolerance, resulting in increased activation of cytotoxic T-cells, T helper cells, B cells, and other immune cells. In a study from a large oncology center in Texas, approximately half of the 100 patients with CPI-induced liver injury had advanced melanoma, and 45% experienced concomitant immune-mediated adverse effects, most commonly dermatological manifestations (14%) and colitis (9%).^41^ In clinical trials, ALT elevations were observed in 3%-15% of patients, and grade III or IV elevations occurred in 2% to 8%.^42^ The risk of DILI was higher with ipilimumab than with other CPIs and was highest with combination therapy using ipilimumab and nivolumab.^42^ Several other risk factors have been identified such as younger age, female sex, and history of immune-mediated disorders.^41^ In a recent US study of 57 patients with a high likelihood of DILI due to CPIs, the implicated agents included pembrolizumab (n = 16), ipilimumab (n = 15), combination of ipilimumab/nivolumab (n = 13), and other CPIs (n = 13). Liver injury developed after a median of 72 days after the first infusion.^43^ HLA alleles associated with autoimmune hepatitis were not overrepresented, in contrast to the findings for many other drugs associated with idiosyncratic DILI. Interestingly, genes associated with the activation of regulatory T cells (*SAMA5A* gene) and 2 host immune response genes (*EDIL3* and *SAMA5A*) and 3 other genes (*GABRP*, *SMAD3*, and *SLCO1B1*) were associated with liver injury due to CPIs although with a relatively low odds ratio (OR: 2.08-2.4, *P* < .01).^43^ In a landmark prospective study from the UK, 38 of 432 patients who received CPIs over 10-year period developed DILI.^44^ The risk of DILI was highest with combination therapy, and no new events occurred after 135 days from the first infusion.^44^ Risk factors for CPI-induced DILI included combination therapy, female sex, higher baseline ALT levels, and lower baseline alkaline phosphatase levels, all of which were independently associated with an increased risk of DILI.^44^ The pattern of liver injury in patients with CPI-induced DILI is heterogeneous. In the recent DILIN study, 53% of patients had hepatocellular injury, 35% had mixed injury, and 15% had cholestatic injury, with younger patients more likely to have hepatocellular injury.^43^ In the UK study, the pattern of liver injury was hepatocellular in 53%, mixed in 29%, and cholestatic in 17% of patients.^44^ In contrast, a recent study from France reported hepatocellular, cholestatic, and mixed patterns of liver injury in 38.5%, 36.8%, and 24.8% of patients, respectively.^45^ Among 117 patients in the French study, liver biopsy was performed in 42%; however, its impact on the diagnosis of DILI was not reported.^45^ The role of liver biopsy in the evaluation of patients with suspected CPI-induced DILI remains controversial. Granulomatous lesions, endothelitis, and lymphocytic cholangitis have been reported,^45^ and microgranulomas and hepatic steatosis were observed in 54% and 46%, respectively, of the 26 liver biopsies in the DILIN study. No pathognomonic histological features of CPI-induced liver injury have been identified, and it remains unclear whether liver biopsy findings alter clinical management.

In a retrospective study from Boston, liver biopsy in patients receiving CPIs who developed moderate-to-severe liver injury delayed the initiation of corticosteroid therapy and was not associated with faster resolution of hepatitis.^46^ However, these findings may be confounded by the indication for liver biopsy. In most patients with liver injury temporally associated with CPI therapy, liver biopsy can likely be avoided if competing etiologies have been excluded through serological testing and imaging, including evaluation for liver metastases. However, liver biopsy may be helpful in atypical cases or in patients who do not respond to corticosteroid therapy and may reduce the need for immunosuppression in selected cases. In a study from Barcelona, an algorithm incorporating temporary discontinuation of CPI therapy and liver biopsy findings avoided corticosteroid therapy in two-thirds of patients with severe DILI.^47^ Although DILI is common in patients receiving CPIs, most patients present with mild-to-moderate liver injury according to international criteria based on measurements of bilirubin and INR.^48^ Jaundice and ALF are uncommon, and death due to hepatotoxicity is also very rare.^47^

Early reports of CPI-induced DILI described predominant hepatocellular injury.^41^ In a recent study from France, mentioned above, cholestatic and mixed patterns accounted for 62% of cases.^45^ In that study, biliary stenosis was identified in 8 patients (6.8%).^45^ This phenomenon, termed sclerosing cholangitis or secondary sclerosing cholangitis (SSC), was first reported in association with CPIs in several publications in 2017.^49^ Secondary sclerosing cholangitis has also been reported in association with idiosyncratic DILI.^50^ Most patients with CPI-induced SSC present with cholestatic or mixed patterns of liver injury and have received anti-PD-1 agents such as pembrolizumab or nivolumab.^51^ Jaundice appears to be more common in patients with SSC, which is associated with a worse prognosis. These patients respond less favorably to corticosteroid therapy than those with other phenotypes of CPI-induced DILI.^52^ In a large retrospective study of approximately 20 000 US veterans, patients who developed immune-related adverse effects (irAEs) had better survival than those who did not.^53^ Among patients with irAEs, those treated with systemic corticosteroids had significantly longer overall survival than those who received steroids for non-irAE-related indications or no corticosteroid therapy (21 months vs. 14 months).^53^

However, among patients who received corticosteroids for irAEs, early corticosteroid use (<2 months after ICI initiation) was associated with a reduced relative survival benefit vs. later steroid use.^53^

Thus, corticosteroid therapy does not appear to abrogate the survival benefit associated with irAEs. However, early corticosteroid use within 2 months of ICI initiation was associated with shorter survival despite continuation of ICI therapy.^53^ However, this might reflect a confounding effect, as the authors pointed out,^53^ because the poorer survival associated with early corticosteroid use and cessation of CPIs might reflect more severe and life-limiting irAEs; however, this hypothesis could be tested in their cohort.

### CDK4/6 Inhibitors

CDK4/6 inhibitors in combination with endocrine therapy have become the standard treatment for endocrine receptor-positive, HER-2-negative metastatic breast cancer. Three agents, ribociclib, palbociclib, and abemaciclib, are widely used for this indication and have recently been associated with hepatotoxicity.

Elevated liver enzyme levels were reported in clinical trials; however, because causality was not systematically assessed and alternative causes of liver injury were not adequately excluded, DILI was not recognized until the postmarketing period. The first published case of ribociclib-induced liver injury originated from France and appeared in 2020; the patient subsequently tolerated treatment with palbociclib.^54^ Shortly after this, 2 cases of ribociclib-induced DILI were reported from Iceland.^55^ Notably, both patients had a distinctive biochemical phenotype characterized by hepatocellular injury with autoimmune features, and liver test results worsened after discontinuation of the drug, necessitating corticosteroid therapy.^55^ Since then, many cases of liver injury associated with the use of cycline-dependent kinases 4 and 6 (CDK4/6) inhibitors have been reported. In a study from France and Belgium, a total of 22 cases of DILI induced by CDK4/6 inhibitors (ribociclib, n = 19 and abemaciclib, n = 3) were identified.^56^ Consistent with the cases reported from Iceland,^55^ approximately 40% of patients had autoimmune features and received corticosteroid therapy to hasten the resolution of DILI.^56^ In a study from Türkiye, hepatotoxicity occurred in 10.5% of patients; consistent with previous reports, hepatocellular injury was the predominant pattern.^57^ Ribociclib was the most frequently implicated agent. No severe consequences of hepatotoxicity, such as ALF, were observed with timely dose reduction or treatment interruption.

### Infliximab

Over the last decade, biological therapies have become an important component of the treatment of autoimmune diseases. The most widely used biologics are the tumor necrosis factor-alpha (TNF-alpha) antagonists, such as infliximab, adalimumab, etanercept, golimumab, and certolizumab pegol. Just as CPIs have revolutionized the treatment of cancer, TNF-alpha antagonists have revolutionized the treatment of many autoimmune disorders. The first reports of infliximab-induced liver injury were unexpected, particularly among rheumatologists with extensive experience using the drug. Because infliximab is a protein that is not metabolized by the liver, it was not anticipated to cause hepatotoxicity. However, infliximab is a well-established cause of liver injury and is the only TNF-alpha antagonists classified as category A in LiverTox, with more than 100 published case reports.^24^ Although adalimumab has recently surpassed infliximab in use in most countries because of similar efficacy and easier administration via the subcutaneous route, adalimumab still belongs to category B in LiverTox, with less than 50 cases reported in the published literature.^24^ The reason for this remains unclear. However, it has been hypothesized that chimeric IgG antibodies of infliximab may appear more foreign to the immune system and that lower serum concentrations of adalimumab following administration may also contribute. In a prospective nationwide DILI study from Iceland, it was found that infliximab together with azathioprine was associated with the highest risk of DILI, with an incidence of 1 case per 148 users of infliximab.^6^ Further studies confirmed the relatively high risk with infliximab-induced liver injury compared with idiosyncratic DILI overall. In a 5-year study, the incidence was approximately 1 case per 120 users of infliximab.^58^ Most patients with infliximab-induced liver injury develop hepatotoxicity after a median of 4 infusions, with hepatocellular injury being the most predominant pattern.^13^ Studies have demonstrated that approximately 50% of patients require corticosteroid therapy because the liver injury is prolonged and may worsen despite discontinuation of infliximab.^13,14,58^ Across multiple studies, most patients with infliximab-induced DILI have been women. In the Icelandic study, 78% of patients with infliximab-induced DILI were female, although infliximab use was similar in men and women.^13^ Likewise, women accounted for approximately 80% of patients in a recent study from the DILIN network.^14^

**Figure 2.**
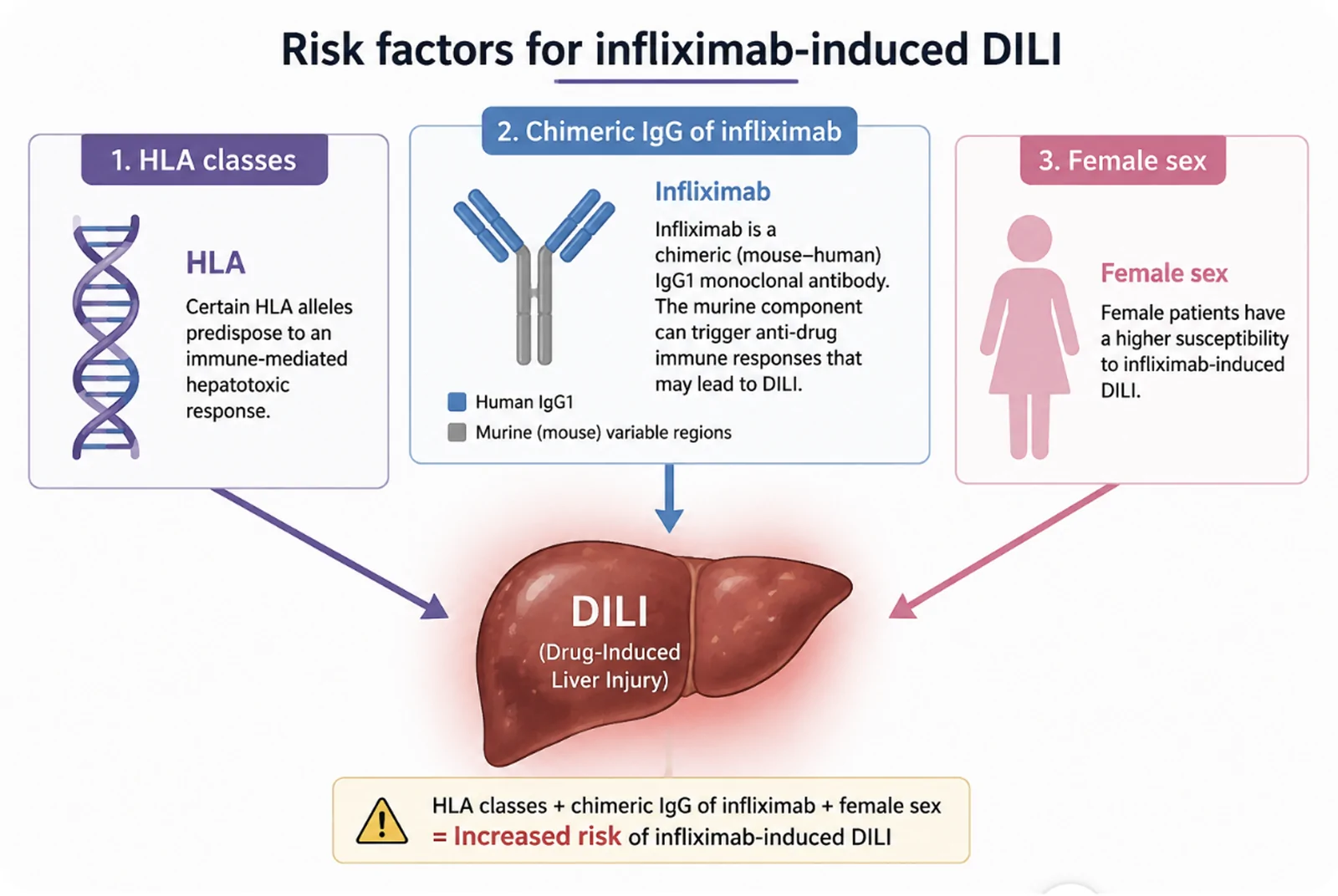
Potential risk factors for infliximab-induced liver injury are demonstrated. These are HLA associations, the chimeric nature of infliximab, and female sex.

Notably, concomitant immunomodulator therapy appears to reduce the risk of infliximab-induced DILI. This effect has been reported for azathioprine^59^ and for methotrexate.^58^ The importance of immunomodulators was confirmed in the recent DILIN study, which showed that approximately 90% of patients with infliximab-induced DILI (n = 27) did not receive a concomitant immunomodulator. This adverse reaction does not appear to represent a class effect, as patients switched to another TNF-α inhibitor have not experienced recurrent hepatotoxicity.^13^ In most published series, outcomes have been favorable.^13^^,^^14^^,^^58^ However, 12 patients have developed ALF due to infliximab, most of whom required emergency liver transplantation.^60^ In a small study of 16 patients with infliximab-induced DILI and 60 controls, Bruno et al identified HLA-B*39:01 as a strong risk factor for infliximab-induced DILI.^61^ Weaker associations were also observed with other HLA alleles.^61^ These findings were not reproduced in a recent population-based study from Iceland.^13^ In that study, 36 patients with infliximab-induced DILI were compared with 50 000 individuals from the general Icelandic population who had previously undergonegenotyping bydeCode genetics* (* www.decode.com *).* The genotypes HLA-DQB1*02:01 and HLA-DRB1*03:01 (odds ratio (OR 2.7, *P* = .013)) were associated with increased risk of infliximab-induced DILI, and HLA-DRB1*04:04 (OR 0.4 [0.14-0.96], *P* = .041) was associated with a decreased risk.^13^ None of the Icelandic patients carried the HLA-B*39:01 allele.^13^ The study by Bruno et al had several methodological limitations, such as a small sample size, few controls, and the absence of corrections for multiple comparisons. HLA associations may reflect a broader genetic predisposition to autoimmune disorders in general, and further studies comparing patients who develop infliximab-induced DILI with infliximab-treated patients who do not are warranted. Figure 2 summarizes potential risk factors for infliximab-induced liver injury.

### Diagnostic Challenges

Although the main objective of the current systematic review is to describe newly identified causative agents, it is worthwhile to mention the important challenges in the diagnosis of idiosyncratic DILI. The art and science of diagnosing this condition have been called the Achilles heel of DILI. Currently, no biomarker can distinguish DILI from other causes of liver injury. Thus, the diagnosis relies on establishing a temporal relationship between exposure to the suspected agent and the onset of liver injury, reasonably excluding competing etiologies, and considering the known hepatotoxic potential of the implicated drug. The differential diagnoses depend on the clinical context. The Roussel Uclaf Causality Assessment Method (RUCAM) provides a structured framework for systematically evaluating the latency and course of liver injury, excluding alternative causes, and assessing the known hepatotoxic potential of the implicated agent.^29^ However, the newly developed revised electronic assessment method (RECAM) offers several important advantages over RUCAM.^62^ These include the elimination of risk factors in RUCAM that are not evidence based and the simplification of latency and dechallenge fields. RECAM has been shown to be better than RUCAM in difficult cases (diagnostic extremes), has increased objectivity, and has increased DILI diagnosis precision.^62^ However, a more accurate causality assessment tool is needed. RECAM is not designed for drugs administered cyclically such as biological therapies and has limited utility when HDSs are suspected. Causality assessment tools also rely on the clinical judgment of experienced hepatologists, an approach referred to as expert opinion.

### Emerging Therapeutic Approaches

The cornerstone of DILI management is discontinuation of the implicated agent(s). Currently, no pharmacological therapy has been proven to alter the natural history of DILI in well-designed, controlled, and/or placebo-controlled trials. Corticosteroid therapy is recommended for patients with DI-ALH^63^ and for those with CPI-induced liver injury, although its use in the latter remains controversial, as discussed earlier. Ursodeoxycholic acid is increasingly used in CPI-used DILI^52^ and other types of idiosyncratic DILI.^64^ However, its use, like that of corticosteroids, is supported primarily by observational studies, and evidence supporting its use remains limited.

## Conclusion

In recent years, several new agents, such as cefazolin and ornidazole, have been recognized as causes of idiosyncratic DILI, with newly recognized phenotypes associated with these widely used antibiotics. Several new HDSs associated with DILI have been identified, such as ashwagandha, *Tinospora cordifolia,* turmeric, and *Garcinia cambogia*, which are the most commonly implicated agents in Western countries. Green tea extract remains the best-documented HDS ingredient, with HLA-B*35:01 identified as a major genetic risk factor. Indirect liver injury associated with the use of CPIs is more common than idiosyncratic DILI, with sclerosing cholangitis emerging as a distinct clinical phenotype. Another class of oncological agents, CDK4/6 inhibitors, has recently been emerged as a well-documented cause of hepatotoxicity. Furthermore, infliximab has a distinct biochemical and clinical phenotype that requires corticosteroids for recovery in approximately 50% of cases. In most patients, the course is mild to moderate; however, more than 10 cases have resulted in emergency liver transplantation. It is of major importance that practicing physicians remain alert to potential liver injury associated with the use of new drugs and HDSs encountered in routine clinical practice.

## Figures and Tables

**Table 1.** Newly Recognized Herbal and Dietary Supplements Associated with Hepatotoxicity: Typical Demographics, Clinical Characteristics, and Outcomes

Herbal and Dietary Supplement	Typical Demographics	Clinical Characteristics of Liver Injury	Outcomes
Polygonum multiflorum (He Shou Wu)	Predominantly middle-aged adults; reported most frequently in Asian populations but increasingly recognized worldwide	Usually hepatocellular injury; latency ranging from days to several months; jaundice common; occasional autoimmune-like features	Most patients recover following withdrawal; severe cases with acute liver failure and liver transplantation have been reported; positive rechallenge well documented
Ashwagandha	Typically middle-aged adults; slight male predominance in some cohorts	Predominantly cholestatic or mixed liver injury with jaundice and pruritus; latency generally 2-12 weeks	Generally self-limited after discontinuation, although prolonged cholestasis may occur; rare cases of acute-on-chronic liver failure and death described
Green tea extract	Most commonly reported in women using weight-loss supplements	Typically hepatocellular injury resembling acute viral hepatitis; relatively short latency; occasional positive rechallenge	Majority recover after cessation; severe hepatotoxicity including acute liver failure and liver transplantation has been reported
Turmeric	More commonly reported in middle-aged and older adults; female predominance observed in several series	Predominantly hepatocellular injury; latency usually 1-4 months; jaundice frequent; association with HLA-B*35:01 described	Most patients recover after withdrawal; rare cases progress to acute liver failure; recurrence after rechallenge reported
*Tinospora cordifolia*	Most reports originate from India; many patients female	Predominantly hepatocellular injury with frequent autoimmune-like phenotype, including elevated IgG and positive autoantibodies	Recovery common after withdrawal with or without corticosteroids; severe liver failure and fatal outcomes have been described
*Garcinia cambogia*	Typically younger or middle-aged adults using weight-loss products; female predominance in many reports	Usually hepatocellular injury with marked aminotransferase elevation; latency typically several weeks to months	Clinical course ranges from complete recovery to acute liver failure requiring liver transplantation
Kratom	Most commonly reported in younger adults; frequently used for pain relief or opioid withdrawal	Typically cholestatic or mixed liver injury; fever and pruritus may occur; latency usually 1-8 weeks	Most patients recover after discontinuation; prolonged cholestasis and rare fatal cases have been reported

IgG, immunoglobulin G.

## Data Availability

The data that support the findings of this study are available on request from the corresponding author.
